# Efficacy of Hermetic Bags in Preserving Canary Beans and Purple Maize Quality in Arequipa, Peru

**DOI:** 10.3390/insects16121240

**Published:** 2025-12-08

**Authors:** Katherine Coronel-Rojas, Dieudonne Baributsa, Sonia J. Zanabria-Galvez, Jorge R. Díaz-Valderrama, Victor H. Casa-Coila

**Affiliations:** 1 Departamento de Ingeniería de Industrias Alimentarias, Universidad Nacional de San Agustín de Arequipa (UNSA), Arequipa 04001, Peru; kcoronelr@unsa.edu.pe (K.C.-R.); szanabriag@unsa.edu.pe (S.J.Z.-G.); 2 Department of Entomology, Purdue University, West Lafayette, IN 47907, USA; jorge.diaz@untrm.edu.pe; 3 Departamento de Agronomía, Universidad Nacional de San Agustín de Arequipa (UNSA), Arequipa 04001, Peru

**Keywords:** grain storage, insect pests, postharvest loss, PICS bags, Latin America

## Abstract

Purple maize and canary beans are economically important crops in Peru, grown for domestic and international markets. However, postharvest storage losses are a major challenge along the value chain due to insect pests. This study evaluated the efficacy of the Purdue Improved Crop Storage (PICS) and polypropylene (PP) bags in storing both crops in the Arequipa region, Peru. Oxygen and CO_2_ levels were monitored every month. Insect pests, germination rates, sugar content, peroxide index, moisture content, and colony-forming units (CFU) were measured at the start and nine months after storage to determine the level of grain deterioration. PICS maintained a hypoxic environment that suppressed insect development and limited grain damage and weight loss to below 1%. In contrast, PP bags allowed infestations to escalate, resulting in weight losses of about 20% for each crop. Germination declined markedly in PP for both crops; in PICS, it remained high for purple maize (87.5%) but fell substantially for canary beans (20.8%). Moisture rose modestly in PICS, yet deterioration markers, including sugar content and peroxide index, were lower than in PP bags. Although *Fusarium*, *Aspergillus*, and *Penicillium* were detected in purple maize across both systems, mold loads were far lower in PICS. Overall, hermetic storage in PICS bags is effective in reducing postharvest losses and preserving important quality characteristics of beans and maize in Peru.

## Simple Summary

Purple maize and canary beans are important commercial crops in Peru but face major postharvest losses from insect pests. This study compared Purdue Improved Crop Storage (PICS) bags with polypropylene (PP) bags for storing both crops over nine months in Arequipa, Peru. We monitored insects, germination, gas levels, sugar content, peroxide index, moisture, and mold load. PICS bags maintained a hypoxic environment that suppressed insects and kept grain weight loss below 1%, compared with about 20% in PP bags. Germination stayed high for purple maize in PICS bags but declined sharply for both crops in PP bags and for canary beans in PICS bags. Although moisture rose slightly in PICS bags, grain quality deterioration was far lower than in PP bags. Overall, PICS bags significantly reduced storage losses and preserved grain quality better than PP bags.

## 1. Introduction

Grains and beans are staple foods, and preserving their quality is crucial for both consumption and maintaining their nutritional value. Quality is strongly influenced by postharvest storage conditions [1]. Globally, postharvest losses are estimated at 30% to 40% of total production [2]. In developing countries, storage accounts for the largest share of postharvest cereal losses, negatively affecting farmers’ livelihoods [3]. When storage conditions are poor, losses can peak, reaching up to 40% in maize [4]. Poor storage also reduces the nutritional content and commercial value of grains [5,6].

Purple maize (*Zea mays* var. *subnigroviolaceo*) is a vital crop for the Andean communities in Peru, valued for its rich ancient heritage, iconic cultural uses, medicinal benefits, and growing export potential [7,8,9,10]. Chicha morada (a purple maize drink) and mazamorra morada (a dessert) are cultural products widely consumed for their high nutritional value. By contrast, canary beans (*Phaseolus vulgaris*) are a cash crop concentrated in the province of Camaná [11] and are sold in national and international markets. For both crops, storage is a major challenge in preserving grain quality.

Grain storage is primarily threatened by high humidity and insect pests. High humidity accelerates grain deterioration and/or loss of seed quality [12,13,14,15]. It also promotes fungal growth, which increases the risk of mycotoxin contamination [16]. Insect pests feed on stored grain, directly degrading quality and causing weight loss that lowers market value. In Peru, insecticides are widely used to control storage pests [11]; however, their reliance raises concerns regarding food safety, the health of applicators, and environmental impacts [17,18,19]. Hermetic storage technologies (e.g., Purdue Improved Crop Storage–PICS–bags) are effective chemical-free alternatives that suppress insect development, minimize damage, and reduce weight loss, thereby preserving grain quality [20,21,22,23].

The objective of the present study was to assess the commercial quality of canary beans and purple maize grains stored in PICS bags and woven polypropylene (PP) bags, which farmers commonly use to store grain. We hypothesize that grains stored in PICS bags will retain higher commercial quality than those stored in PP bags. To our knowledge, no studies have evaluated hermetic bags for grain storage in Peru, nor have they specifically assessed their use for purple maize and canary beans. This study provides the first crop- and context-specific evidence to guide stakeholders in integrating hermetic storage into Peru’s grain value chain.

## 2. Materials and Methods

### 2.1. Pest Identification in Field Samples

Preliminary pest identification was conducted using grain samples collected from local fields. An additional pest assessment was done during the storage experiment.

### 2.2. Storage Experimental Setup

This study was conducted in Camaná (16°37′11.4″ S, 72°42′48.4″ W) and Castilla (16°10′26.8″ S, 72°28′43.2″ W) provinces, the main producing areas of canary beans and purple maize, respectively, in the department of Arequipa, Peru [11,24]. Storage trials ran from 17 November 2019 to 22 August 2020 for canary beans and from 9 January 2020 to 8 October 2020 for purple maize. These experiments were originally designed to run for six months, which reflects typical on-farm storage duration, but were extended to nine months because COVID-19 travel restrictions and lockdowns prevented earlier access. Naturally infested, non-insecticide-treated grains of canary beans and purple maize ears were sourced from local farmers. The initial moisture content was 9.8% for purple maize and 12.8% for canary beans. A completely randomized design was used, with two storage types as treatments (Purdue Improved Crop Storage–PICS and Polypropylene–PP–bags) and four replicates for each. The 50 kg PICS bags were obtained from Purdue University (West Lafayette, IN, USA), while the 50 kg PP bags were purchased from local markets in Arequipa, Peru. Each bag (experimental unit or replicate) was filled with 22 kg of purple maize or canary beans. The PICS and PP bags were stored under ambient conditions in local warehouses, with bean bags kept in Camaná and maize bags kept in Castilla.

### 2.3. Data Collection

#### 2.3.1. Pest Identification in Field Samples

Before the experiment, grain field samples were collected to identify storage insect pests. Canary bean samples were taken from the districts of Camaná, José María Quimper, Mariscal Cáceres, Nicolás de Piérola, Ocoña, and Samuel Pastor in the province of Camaná. Purple maize samples were collected from the districts of Huancarqui, Aplao, and Uraca in the province of Castilla. For each crop, 50 agricultural fields were sampled. From each field, dried bean plants or maize ears were collected, threshed or shelled, and cleaned before storage. A 500 g grain sub-sample from each field was placed in 500 mL PET bottles covered with mesh clothes to allow airflow. These samples were stored under ambient conditions in both locations (11 months for beans in Camaná and 9 months for purple maize in Castilla) to simulate typical farmers’ storage environments. After storage, all 50 samples (500 g each) per crop were examined to identify the insect pest species present.

#### 2.3.2. Storage Experiment

During the experiment, we evaluated oxygen and carbon dioxide, live and dead insects, damaged grain, weight loss, moisture content, sugar content, peroxide index, germination, and postharvest pathogens (as measured by colony-forming units). Except for O_2_ and CO_2_ levels, all data were collected at the start and after nine months of storage. For canary beans, samples were randomly collected from each bag (replicate) by pushing a locally made compartmentalized spear probe from top to bottom from the four cardinal points, homogenized, and three 200 g composite sub-samples were subsequently obtained for parameter evaluation. For purple maize, in each replicate, ears were randomly selected from the upper, middle, and lower sections of the bags, shelled, and the resulting grains homogenized prior to the collection of three 200 g sub-samples for further analysis.

*(i)*  Oxygen (O_2_) and carbon dioxide (CO_2_): The Mocon Pac Check^®^ 325 device (Mocon, Minneapolis, MN, USA) was used to measure the O_2_ and CO_2_ levels in each replicate of the PICS and PP bags every month. This device measures the O_2_ and CO_2_ composition of a closed bag by extracting a small sample of air from the inside environment. The Mocon device uses a 20-gauge hypodermic needle to sample the gas inside the bag. For the PICS bag measurement, the outer bag was opened, and the inner liners were pierced near the top with the analyzer needle. The punctures were sealed with 10 mm adhesive pads and reinforced with packing tape after each reading. For subsequent measurements, the same site was accessed by briefly unsealing and then resealing the adhesive tape. PP bag measurements were taken at the same spots without sealing, as they are not airtight.
*(ii)*  Relative Humidity and Temperature: Relative humidity and temperature were recorded using EasyLog EL-USB-2 USB data loggers (Lascar, Erie, PA, USA). Data were recorded every 6 h throughout the storage period. Due to the limited number of data loggers, one was placed in a single replicate of each treatment and in the room where the experiments were conducted (ambient) to monitor environmental conditions inside and outside of the bags during the experiment.
*(iii)*  Live and dead insects: We followed [25] to assess the total count. We counted live and dead insects in three 200 g sub-samples per replicate (12 sub-samples per treatment).
*(iv)*  Damaged and undamaged grains: Three 200 g sub-samples per replicate (12 per treatment) were used to measure the weight of undamaged (Wu) and damaged (Wd) grains by insects and the number of undamaged (Nu) and damaged (Nd) grains by insects. The percentage of damage was calculated according to [26]:
  $$Damage\left(\%\right)=\left[\frac{Nd}{\left(Nd+Nu\right)}\right]\times 100$$
*(v)*  Weight loss (%): It was calculated using the count and weight method [27]:
  $$Weight\;loss\left(\%\right)=\left[\frac{\left(Wu*Nd-Wd*Nu\right)}{Wu\left(Nu+Nd\right)}\right]\times 100$$
*(vi)*  Seed germination: One hundred seeds in good phytosanitary condition were selected from each replicate and divided into four sub-samples of 25 seeds. Each sub-sample was placed in a Petri dish lined with filter paper moistened with sterile water; moisture was maintained throughout the test. Seeds were examined daily. A seed was considered germinated when the radicle reached 1–2 cm in length. The test concluded after five days, at which point germinated and non-germinated seeds were counted. To determine the germination percentage (GP), we used the following formula:
  $$GP=\frac{seed\;germinated}{total\;seeds}\times 100$$
*(vii)*  Moisture content: Moisture content of the grains was determined according to the NTP 205.002 1979—Rev. 2016 [28] using the oven-drying method. The procedure involved grinding the grain so that 99% of particles passed through a 0.841 mm sieve. A 5 g sample was collected from each of the 200 g sub-samples, weighed, and dried in a forced-air oven at 130 °C for 60 min, cooled in a desiccator, and reweighed. The moisture content was then calculated as the percentage of weight loss relative to the initial sample mass.
*(viii)*  Sugar content and peroxide index: Sugar content was determined following the method described by [29]. Grain samples (200 g) were hydrolyzed, and sugar content was quantified using Fehling’s solution titration. During the procedure, a prepared sample solution was titrated against a mixed Fehling solution under heat, with methylene blue as the indicator. The appearance of a bright red copper oxide precipitate identified the endpoint. The peroxide index was determined according to NTP 209.006 [30], which measures lipid oxidation. Ground grain samples were dissolved in acetic acid and chloroform, then treated with potassium iodide solution. The released iodine was titrated with standardized sodium thiosulfate using starch as an indicator.
*(ix)*  Mold Colony-Forming Units: Fungal incidence was assessed only in purple maize due to its high susceptibility to mold under elevated moisture. Total mold counts were performed following the ICMSF serial dilution protocol (2000) at BHIOS Laboratories (https://bhioslabs.com/). A serial dilution of 10 g of grains collected from each replicate was performed on peptone water and plated in duplicates on Oxytetracycline–Glucose Yeast Extract Agar (OGYE) culture medium. The samples were incubated at 25 °C for 5 days in the dark. Mold and yeast colonies on each plate were observed, differentiated, and identified following the laboratory’s quality control protocols. The results were expressed in colony-forming units (CFU)/g. We made observations of colonies to determine the genus to which the mold belongs, following descriptive illustrations from [31]. These observations were made using an optical microscope, Primostar (Zeiss, Oberkochen, Germany).

### 2.4. Statistical Analysis

The data were analyzed using the IBM Statistical Package for Social Science (SPSS) software, version 30. Each bag served as the experimental unit, and for some variables, three sub-samples were collected per bag to characterize within-bag variability. A one-way ANOVA followed by Tukey’s HSD test was used to compare means across the four treatment–time combinations (PICS vs. PP × 0 vs. 9 months) to provide a unified comparison. When normality assumptions were violated, non-parametric analyses were applied. Differences among the four treatment–time groups were assessed using the Kruskal–Wallis test. For oxygen and carbon dioxide data, mean concentrations were compared across months during the storage duration. Means ± SEM were separated at *p* < 0.05.

## 3. Results

### 3.1. Insect Identification

Analysis of the 50 purple maize field samples showed that 52% were infested. Among the infested samples, *Sitotroga* sp. was present in 100%, *Sitophilus* sp. in 96.2%, *Pagiocerus* sp. in 11.5%, *Tribolium* sp. in 7.7%, and *Cryptolestes* sp. in 7.7%, with *Sitotroga cerealella* (Olivier) and *Sitophilus* being the most prevalent species. In canary beans, 58% of samples were infested, and all contained a single species: *Acanthoscelides obtectus* (Say).

### 3.2. Oxygen and Carbon Dioxide

**Figure 1:**
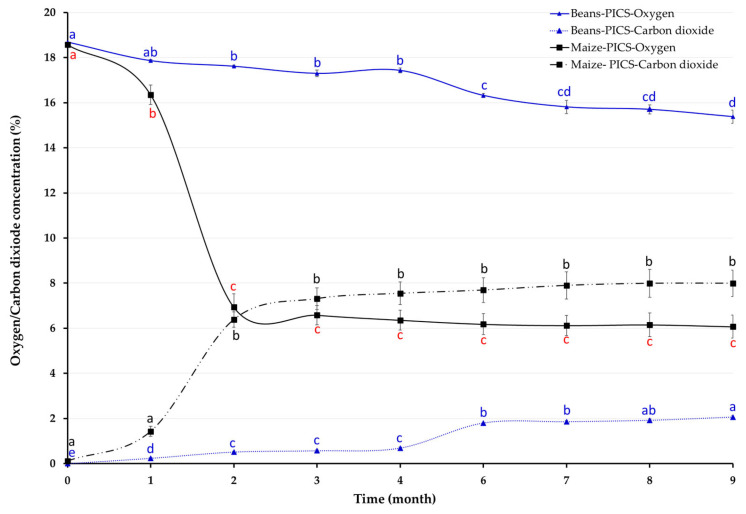
Oxygen and carbon dioxide concentration inside Purdue Improved Crop Storage (PICS) bags storing canary beans in Camaná and purple maize in Castilla for nine months, Arequipa region, Peru. Within each crop (line), monthly means of O_2_ or CO_2_ concentrations followed by different letters differ significantly at *p* < 0.05.

Oxygen and CO_2_ changed during storage in both crops in PICS bags (Figure 1). ANOVA revealed significant effects of storage time on oxygen levels (maize: F_(8, 27)_ = 118.40, *p* < 0.001; beans: F_(8, 18)_ = 44.86, *p* < 0.001) and carbon dioxide (maize: F_(8, 27)_ = 40.90, *p* < 0.001; beans: F_(8, 18)_ = 414.10, *p* < 0.001). Oxygen declined over time, but the decrease was more pronounced in PICS containing purple maize than in those with canary beans. After nine months, O_2_ and CO_2_ levels were 6.07% and 7.99% for purple maize and 15.38% and 2.06% for canary beans, respectively. Unlike in PICS bags, gases in PP bags remained essentially unchanged, fluctuating between 17.84 and 18.54% for O_2_ and 0.00–0.06% for CO_2_.

### 3.3. Relative Humidity and Temperature

**Figure 2:**
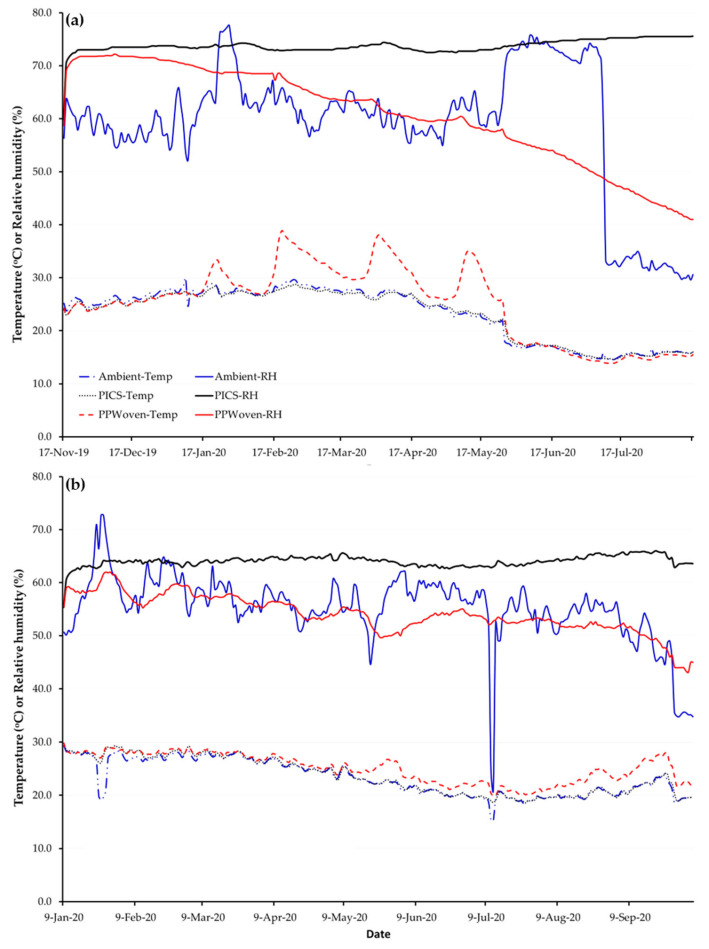
Average daily temperature and relative humidity in the room (ambient), inside Purdue Improved Crop Storage (PICS) and polypropylene (PP) bags storing (**a**) canary beans in Camaná and (**b**) purple maize in Castilla for nine months, Arequipa region, Peru.

Relative humidity (RH) and temperature in the storage room and inside PICS and PP bags varied over time (Figure 2). For purple maize in Castilla, temperatures in PICS bags exhibited smaller diurnal swings than in PP bags. Ambient and PP bag RH oscillated strongly and drifted seasonally, while it remained stable in PICS bags. Similarly, for canary beans stored in Camaná, RH in PICS bags was more stable relative to the ambient air and in PP bags. Unlike in Castilla, RH in PP bags in Camaná showed slight fluctuation and declined over time relative to ambient conditions.

### 3.4. Grain Infestation and Quality

**Figure 3:**
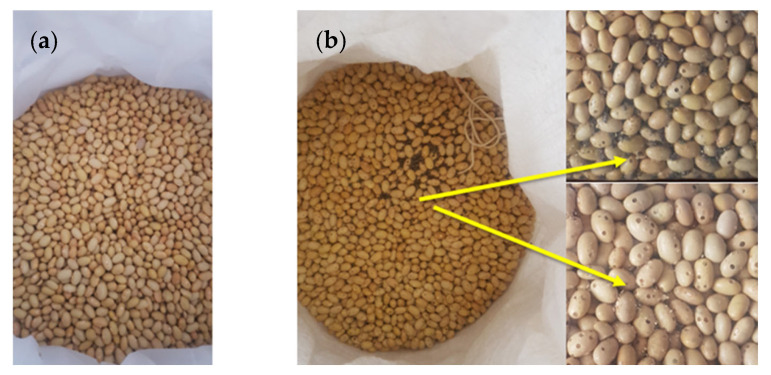
Canary beans stored for nine months in (**a**) Purdue Improved Crop Storage (PICS) and (**b**) polypropylene (PP) bags in Camaná, Arequipa region, Peru. Arrows show damaged beans.

**Figure 4:**
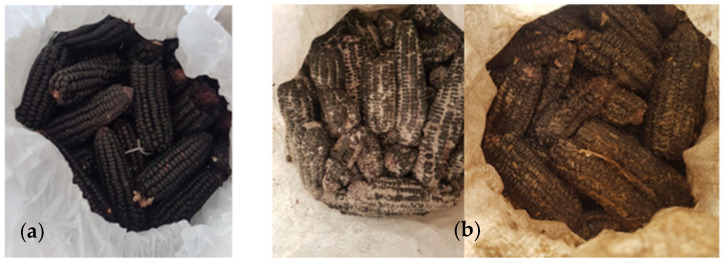
Purple maize stored for nine months in (**a**) Purdue Improved Crop Storage (PICS) and (**b**) polypropylene (PP) bags in Castilla, Arequipa region, Peru.

During storage experiments, *Sitophilus zeamais* and *A. obtectus* were the main pests of purple maize and canary beans, respectively. There were significant differences among treatments in grain damage (maize: F_(3, 44)_ = 196.78, *p* < 0.001; beans: H_(3)_ = 27.51, *p* < 0.001), grain weight loss (maize: F_(3, 44)_ = 4.28, *p* < 0.009; beans: H_(3)_ = 30.61, *p* < 0.001), number of live insects (maize: H_(3)_ = 45.74, *p* < 0.001; beans: H_(3)_ = 43.36, *p* < 0.001), and number of dead insects (maize: H_(3)_ = 39.69, *p* < 0.001; beans: H_(3)_ = 41.59, *p* < 0.001). Initially, both storage methods performed similarly, but after nine months, PICS bags showed far lower infestation, damage, and weight loss than PP bags (Table 1; Figure 3 and Figure 4). No live insects were found in PICS bags, whereas PP bags contained heavy infestations. Dead insects were minimal in PICS bags but high in PP bags.

### 3.5. Germination

Data analysis showed significant differences among treatments in germination (maize: H_(3)_ = 13.12, *p* = 0.004; beans: H_(3)_ = 13.79, *p* = 0.003). Initially, germination rate averaged 96.88% for purple maize and 99.75% for canary beans (Table 2). After nine months, germination declined in both bag types but to very different degrees. In purple maize, the drop was nine percentage points in PICS bags versus 77 percentage points in PP bags; in canary beans, the declines were 79.25 percentage points in PICS and 85.5 percentage points in PP bags. These reductions were statistically significant for canary beans in both bag types and for purple maize in PP bags, but not for purple maize in PICS.

### 3.6. Physicochemical Assessment

Statistical analysis revealed significant differences among treatments in grain moisture content (maize: F_(3, 12)_ = 374.37, *p* < 0.001; beans: F_(3, 12)_ = 425.85, *p* < 0.001), sugar content (maize: H_(3)_ = 13.29, *p* = 0.004; beans: H_(3)_ = 12.55, *p* = 0.006), and peroxide index (maize: H_(3)_ = 13.18, *p* = 0.004; beans: H_(3)_ = 14.33, *p* = 0.002). Moisture levels increased in PICS bags relative to PP bags and the initial in both crops (Table 3). Sugar content significantly increased in purple maize but declined in canary beans, regardless of the storage type (PICS or PP bags–Table 3). The peroxide index of purple maize stored in PICS was unchanged compared with the initial values, whereas it increased slightly in canary beans (Table 3). In contrast, peroxide levels increased from the initial in both crops stored in PP bags.

### 3.7. Incidence of Postharvest Pathogens

At the initial (0 month), mold (phytopathogen) in purple maize analysis was 418.75 ± 2.06 CFU/g for PICS bags and 450.5 ± 1.71 CFU/g for PP bags, respectively. However, after 9 months, there was a significant increase: PP bags reached 9333.33 ± 480.74 CFU/g, compared to 1400 ± 548.77 CFU/g in PICS bags. Purple maize samples showed the presence of *Fusarium* sp., *Aspergillus* sp., and *Penicillium* sp. in both storage systems. Significant differences were observed between the two treatments (F_(3, 11)_ = 131.28, *p* < 0.001).

## 4. Discussion

Postharvest losses in maize and common beans are substantial, primarily driven by storage insects. In this study, initial infestations were low but increased sharply in PP bags, resulting in significant grain damage and weight loss. In contrast, PICS bags suppressed insect development and limited losses to below 1% in both purple maize and canary beans. This suppression is driven by hypoxic conditions that form inside airtight containers [32,33]. Although O_2_ levels in PICS bags did not fall to the typical 5% threshold for insect mortality, the sustained low levels still suppressed pest growth and feeding, greatly reducing grain damage. These findings align with previous studies demonstrating that hermetic bags effectively preserve grain quality in maize and common beans [34,35,36,37,38,39,40,41].

The insect species detected in both crops were typical of those found in stored maize and common beans [42,43,44,45]. The higher post-storage insect mortality in canary beans likely reflects species-specific differences in adult longevity: *Sitophilus zeamais* adults can survive for several months, whereas *A. obtectus* typically lives only about two weeks [46,47,48]. These differences in lifespan may explain the greater number of live adults recorded in purple maize stored in PP bags and the higher adult mortality observed in canary beans kept in PP bags.

Seed germination responses differed by crop and storage method. In PP bags, germination declined sharply for both purple maize and canary beans due to insect feeding and fluctuating humidity. In PICS bags, purple maize maintained high seed viability because moisture content and relative humidity remained within safe storage ranges. In canary beans, however, germination still declined despite minimal insect pressure. This reduction appears driven by consistently high relative humidity inside PICS bags—above the ~70% threshold known to accelerate loss of seed viability even under hermetic storage [49]. This outcome aligns with evidence that elevated seed moisture and high relative humidity impair germination and viability during storage, including in hermetic systems [13,50,51,52]. Hermetic storage can preserve seed quality when moisture levels are appropriate, in part because it stabilizes the microenvironment—especially relative humidity—which strongly influences seed viability [35,43,53,54,55,56].

Sugar content in purple maize rose under both storage systems but rose much more in PP, indicating greater deterioration from starch hydrolysis driven by warmth, time, and fungal activity [57,58,59]. Because higher sugar content correlates with reduced vigor and quality, this pattern aligns with the low germination observed in PP bags [60]. In contrast, purple maize in PICS had a lower peroxide index, reflecting reduced lipid oxidation under low-oxygen conditions. This is consistent with evidence that oxygen-permeable packaging (e.g., PP bags) increases oxidation [61,62,63]. In canary beans, the slight increase in peroxide index in both PP and PICS bags suggests only modest overall oxidation.

After nine months, postharvest fungi—mainly *Fusarium*, *Aspergillus*, and *Penicillium*—were detected in purple maize under both storage systems. These genera commonly colonize stored maize and include species capable of producing mycotoxins [12,64,65,66,67]. Although these common storage fungi include mycotoxin-producing species, PICS bags limited fungal proliferation and the associated quality and nutritional value loss compared with PP bags. This aligns with studies showing that when initial moisture is above acceptable levels, mold growth and aflatoxin contamination increase in PP and jute bags but remain low in PICS bags [50,65,68,69,70,71].

## 5. Conclusions

Overall, this research demonstrated that hermetic bags are effective in preserving the quality of commercial crops, such as purple maize and canary beans, in Peru. Hermetic bags suppressed insect development, grain damage, and hence weight loss. In addition, hermetic bags preserve the germination of purple maize but not that of canary beans. Environmental conditions such as high moisture and relative humidity contributed to the degradation of canary bean quality stored in hermetic bags. Canary beans should be dried to a safe moisture content (below 12%) before storage in hermetic bags. Fungal growth was minimal in purple maize stored in PICS bags but increased significantly in PP bags. An important limitation of this study is that mycotoxin levels (e.g., aflatoxins, fumonisin) were not measured due to resource and analytical constraints. Future research should therefore include systematic monitoring of mycotoxin accumulation in purple maize during storage, assessing germination of low-moisture canary beans stored in PICS bags, and evaluating the economic viability of PICS by comparing its costs and benefits with conventional storage. Together, these studies would clarify the biological, food safety, and economic impacts of PICS for both crops.

## Figures and Tables

**Table 1:** Average (±standard error of mean, SEM) grain damage and weight loss, and live and dead insects in purple maize and canary beans stored for nine months in Purdue Improved Crop Storage (PICS) and polypropylene (PP) bags in the Arequipa region, Peru.

Variable	Sampling Time	Purple Maize *		Canary Beans	
		PICS Bags	PP Bags	PICS Bags	PP Bags
Damage (%)	Initial	0.66 ± 0.09 b	0.63 ± 0.11 b	0.31 ± 0.07 b	0.21 ± 0.06 b
	9 months	0.85 ± 0.13 b	87.16 ± 6.16 a	1.34 ± 0.54 b	82.39 ± 1.19 a
Weight loss (%)	Initial	0.24 ± 0.03 b	0.31 ± 0.05 b	0.06 ± 0.02 b	0.10 ± 0.03 b
	9 months	0.25 ± 0.04 b	19.87 ± 10.22 a	0.41 ± 0.22 b	19.27 ± 4.08 a
Live insects	Initial	0.0 ± 0.0 b	0.0 ± 0.0 b	0.0 ± 0.0 b	0.1 ± 0.1 b
	9 months	0.0 ± 0.0 b	77.2 ± 10.1 a	0.0 ± 0.0 b	4.8 ± 0.7 a
Dead insects	Initial	0.0 ± 0.0 b	0.0 ± 0.0 b	0.0 ± 0.0 b	0.0 ± 0.0 b
	9 months	0.4 ± 0.2 b	9.1 ± 1.2 a	0.3 ± 0.2 b	177.3 ± 7.1 a

* Under the same crop and within the same variable, across sampling time and storage type (PICS or PP bags), means followed by different letters differ significantly at *p* < 0.05.

**Table 2:** Average (±standard error of mean, SEM) germination (%) of purple maize and canary beans stored for nine months in Purdue Improved Crop Storage (PICS) and polypropylene (PP) bags in the Arequipa region, Peru.

Sampling Time	Purple Maize *		Canary Beans	
	PICS Bags	PP Bags	PICS Bags	PP Bags
Initial	96.50 ± 0.50 a	97.25 ± 0.85 a	100.00 ± 0.00 a	99.50 ± 0.29 a
9 months	87.50 ± 1.85 a	20.25 ± 20.25 b	20.75 ± 0.85 b	14.00 ± 1.87 c

* Under the same crop, across sampling time and storage type (PICS or PP bags), means followed by different letters differ significantly at *p* < 0.05.

**Table 3:** Average (± standard error of mean, SEM) moisture, sugar content, and peroxide index of purple maize and canary beans stored for nine months in Purdue Improved Crop Storage (PICS) and polypropylene (PP) bags in the Arequipa region, Peru.

Variable	Sampling Time	Purple Maize *		Canary Beans	
		PICS Bags	PP Bags	PICS Bags	PP Bags
Moisture (%)	Initial	9.77 ± 0.03 c	9.77 ± 0.02 c	12.78 ± 0.06 b	12.77 ± 0.05 b
	9 months	12.64 ± 0.08 a	10.39 ± 0.11 b	14.13 ± 0.11 a	9.88 ± 0.11 c
Sugar content (%)	Initial	0.76 ± 0.00 c	0.78 ± 0.01 c	0.07 ± 0.00 a	0.08 ± 0.00 a
	9 months	2.45 ± 0.08 b	3.37 ± 0.15 a	0.03 ± 0.00 b	0.04 ± 0.00 b
Peroxide index (mEq/kg)	Initial	0.48 ± 0.00 b	0.48 ± 0.00 b	0.27 ± 0.01 b	0.27 ± 0.00 b
	9 months	0.72 ± 0.06 b	1.23 ± 0.12 a	0.30 ± 0.00 a	0.30 ± 0.00 a

* Under the same crop and within the same variable, across sampling time and storage type (PICS or PP bags), means followed by different letters differ significantly at *p* < 0.05.

## Data Availability

The original contributions presented in the study are included in the article; further inquiries can be directed to the corresponding authors.
